# Comparison of bronchial hygiene techniques in mechanically ventilated patients: a randomized clinical trial

**DOI:** 10.5935/0103-507X.20190005

**Published:** 2019

**Authors:** Wagner da Silva Naue, Bruno Barcelos Herve, Fernando Nataniel Vieira, Gracieli Nadalon Deponti, Luciane de Fraga Martins, Alexandre Simões Dias, Silvia Regina Rios Vieira

**Affiliations:** 1 Hospital de Clínicas de Porto Alegre, Universidade Federal do Rio Grande do Sul - Porto Alegre (RS), Brasil.

**Keywords:** Physical therapy modalities, Insufflation, Ventilators, mechanical, Hygiene, Bronchoalveolar lavage, Respiratory aspiration

## Abstract

**Objective:**

To compare the effects of vibrocompression and hyperinflation with mechanical ventilator techniques alone and in combination (hyperinflation with mechanical ventilator + vibrocompression) on the amount of aspirated secretion and the change in hemodynamic and pulmonary parameters.

**Methods:**

A randomized clinical trial with critically ill patients on mechanical ventilation conducted in the intensive care unit of a university hospital. The patients were randomly allocated to receive one of the bronchial hygiene techniques for 10 minutes (vibrocompression or hyperinflation with mechanical ventilator or hyperinflation with mechanical ventilator + vibrocompression). Afterwards, the patients were again randomly allocated to receive either the previous randomly allocated technique or only tracheal aspiration. The weight of aspirated secretions (in grams), ventilatory mechanics and cardiopulmonary data before and after the application of the techniques were analyzed. The tracheal reintubation frequency and time and mortality on mechanical ventilation were also evaluated.

**Results:**

A total of 93 patients (29 vibrocompression, 32 hyperinflation with mechanical ventilator and 32 hyperinflation with mechanical ventilator + vibrocompression) on mechanical ventilation for more than 24 hours were included. The hyperinflation with mechanical ventilator + vibrocompression group was the only one that presented a significant increase in aspirated secretions compared to tracheal aspiration alone [0.7g (0.1 - 2.5g) *versus* 0.2g (0.0 - 0.6g), p value = 0.006].

**Conclusion:**

Compared to tracheal aspiration alone, the combination of hyperinflation with mechanical ventilator + vibrocompression techniques was most efficient for increasing the amount of aspirated secretions.

## INTRODUCTION

Patients admitted to intensive care units (ICUs) present with altered pulmonary secretion clearance and production, changes in mucociliary transport and bronchial hypersecretion. The hypersecretion is due to the action of inflammatory mediators and an increase in the number and excretion of mucous glands.^(^^1-4^^)^ Changes in mucociliary transport may occur due to the presence of the orotracheal tube, periods of hypoxemia, dehydration, inadequate humidification of the ventilated air and bed rest, which are common circumstances for critically ill patients on mechanical ventilation (MV).^(^^2,5^^)^

The aim of MV is to reduce the ventilatory work and maintain gas exchange,^(^^3,4^^)^ but it also has deleterious effects on mucociliary transport and coughing ability.^(^^1,2^^)^ These effects provoke the stasis of secretions in the airways and bronchial obstruction,^(^^3,5^^)^ with hypoventilation, atelectasis and consequent hypoxemia. This set of factors also favors microorganism multiplication and, thus, an increased incidence of ventilator-associated pneumonia (VAP).^(^^6,7^^)^

To reverse or reduce these deleterious effects, bronchial hygiene techniques are used by physical therapists in several ICUs around the world. Among these techniques, tracheal aspiration, vibrocompression (VB) and hyperinflation with mechanical ventilation (HMV) are commonly employed. They can be used separately or in combination according to the pathology and patient clinical status.^(^^8-10^^)^ However, the effect of these techniques on patients on MV remains unclear since the hypothesized increase in the amount of pulmonary secretions aspirated after their application has not yet been confirmed.^(^^11,12^^)^ In addition, methodological differences among studies and the application of combined techniques make it impossible to evaluate the effect of each technique on the amount of aspirated pulmonary secretions.^(^^13^^)^

The aim of this study is to evaluate the amount of aspirated pulmonary secretions in critically ill patients on MV before and after the individual application of three different bronchial hygiene techniques: VB, HMV and VB combined with HMV (VB + HMV). An additional objective was to compare these techniques to tracheal aspiration alone by evaluating the hemodynamic and pulmonary effects, frequency of tracheal reintubation, and the time and mortality on MV.

## METHODS

**Figure 1:**
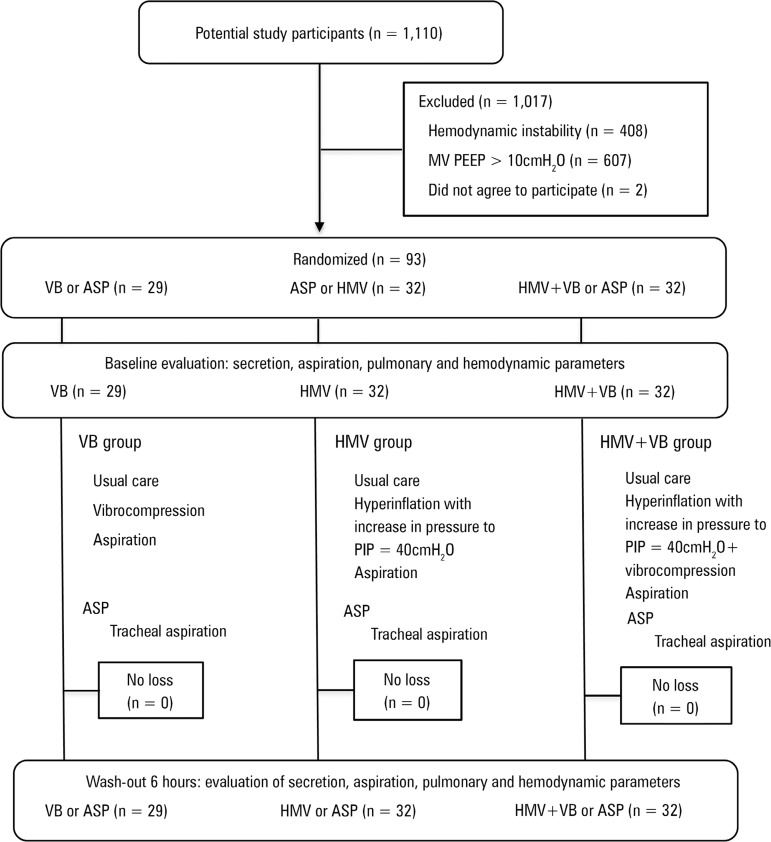
Study design flowchart. MV - mechanical ventilation; PEEP - positive end expiratory pressure; VB - vibrocompression; ASP - pulmonary aspiration alone; HMV - hyperinflation with mechanical ventilator; PIP - peak inspiratory pressure.

A randomized controlled trial was conducted at the level four general ICU of a university hospital in the city of Porto Alegre, Rio Grande do Sul, Brazil, and registered with clinicaltrials.gov under the identifier NCT 02604082. The study was reported according to the CONSORT protocol^(^^14^^)^ (Figure 1). Participants and all collection assistants except the trained physical therapists (who had 5 to 12 years of experience in intensive care) were blinded to the technique application groups and the results of the secretion collections.

The study included patients older than 18 years who were admitted to the ICU, were on MV for a maximum of 72 hours, and who met the following inclusion criteria: positive end expiratory pressure (PEEP) ≤ 10cmH_2_O and hemodynamically stability, with mean arterial pressure (MAP) ≥ 60mmHg and norepinephrine doses ≤ 0.5µg/kg/minute. Patients with contraindications to increased positive pressure (subcutaneous emphysema, undrained pneumothorax and hemothorax); rib fractures; obesity (body mass index (BMI) ≥ 35); the need for MV with peak pressures ≥ 40cmH_2_O; and those diagnosed with osteoporosis and acute respiratory distress syndrome were excluded from the study. An informed consent form was provided and completed by the patients' guardians. The study was conducted as defined by the Ethics Committee responsible for human studies at the *Hospital de Clínicas de Porto Alegre* (HCPA), *Universidade Federal do Rio Grande do Sul* - UFRGS) 11-0367, according to the Declaration of Helsinki of 1975, revised in 2000.

The data collected were age, sex, clinical diagnosis at admission, Acute Physiological and Chronic Health Evaluation II (APACHE II) score, heart rate (HR), respiratory rate (RR), MAP and peripheral arterial oxygen saturation (SpO_2_) measured with a Philips^®^ multiparameter monitor (IntelliVue MP60, Philips Medical Systems, São Paulo, Brazil); peak inspiratory pressure (PIP), PEEP, auto-PEEP and tidal volume (TV), measured with a mechanical ventilator (Servo-s^®^ or Servo-I^®^, Maquet); and dynamic compliance (Cdyn, calculated as TV/PIP-PEEP). To compare time points and groups, variations in the respective parameters at each moment were considered (Δ = final value minus initial value). Additionally, the time on MV, mortality during MV, and the need for tracheal reintubation within 48 hours were recorded.

The allocation of patients to the different study groups and the initial technique to be used (aspiration alone or an investigated technique) were randomized by the computer program Randomization (http://www.randomization.com) in block format with eight subjects per group. After randomization, patients were included in the groups following the list generated by a blinded collaborator.

After randomization, the patients were allocated into three groups: the VB group, which was subjected to VB only; the HMV group, which was subjected to HMV only; and the VB + HMV group, which was subjected to VB combined with HMV. In addition, the patients were randomized again for the initial application of pulmonary aspiration (ASP) alone or the investigated technique (VB or HMV or VB + HMV). The MV of the three groups of patients during the application of techniques was adjusted to the assisted-controlled (A/C) pressure-cycled ventilation (PCV) mode with a RR of 12 breaths per minute and an inspiration-to-expiration ratio of 1:2. VB was performed by eccentric isometric contraction of the upper limbs by the physical therapist to produce vibration and was combined with compression of the patient's chest in the expiratory phase. HMV was performed in the PCV mode with an increase in the initial positive inspiratory pressure until it reached a peak pressure of 40cmH_2_O.^(^^9,10^^)^ The techniques were applied for 10 minutes at a time twice a day during the time that the patients remained on MV.

After being allocated to the groups and randomized to the initial technique that was used, all patients were placed in dorsal decubitus with the head elevated 30º. They were then aspirated only once, with a number 12 probe (MarkMed^®^ indústria e comércio LTDA, São Paulo, Brazil) and a vacuum pressure of -40cmH_2_O; this time point was considered the baseline aspiration and was used to match the groups in terms of pulmonary secretion volume. After 2 hours, hemodynamic and pulmonary parameters were collected and recorded. Next, depending on the randomization, the patients received either one of the studied techniques or ASP. Secretion collection involved open system aspiration three times for 12 seconds at an interval of 30 seconds, with the same probe size and vacuum pressure applied for all three groups. The aspirated secretions were stored in a collection flask (Intermedical^®^; Intermedical - Setmed, São Paulo, Brazil). The hemodynamic and pulmonary parameters were collected again 1 minute after the aspirations.

After a 6-hour wash-out period, all the patients were positioned and aspirated once time in the same manner used for the baseline aspiration. After 2 hours, the hemodynamic and pulmonary parameters were collected and recorded before the appropriate techniques were applied; patients who were randomly allocated to receive the study technique first (VB, HMV or VB + HMV) received only ASP at this time, while those who had received only ASP first received one of the study techniques (VB, HMV, VB + HMV) at this time. Secretions were again aspirated (using the open system) three times for 12 seconds with an interval of 30 seconds between each aspiration and using the same probe number and vacuum pressure that were used previously; the secretions were then collected and stored. Hemodynamic and pulmonary parameters were collected again and recorded 1 minute after application of the studied techniques.

**Figure 2:**
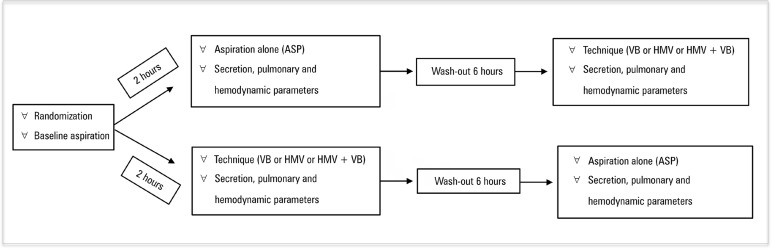
Application protocol of the study. ASP - pulmonary aspiration alone; VB - vibrocompression; HMV - hyperinflation with mechanical ventilator.

The secretions aspirated to the collection flasks at each time point (ASP, VB, HMV and HMV + VB) were weighed on a precision scale (model MSA 524P-000-DA, Sartoriu Cubis^®^, Frankfurt, Germany) in the microbiology laboratory of HCPA by a collaborator who was blinded to the study. At the time of collection, any secretions remaining in the probe were not considered, and only the secretions in the collection vial were weighed. The study protocol is described in figure 2.

The sample size was calculated considering the mean and standard deviation values of the amounts of aspirated pulmonary secretions reported in previous studies^(^^6^^)^ and clinically significant minimum values. The mean was a difference of 0.75 ± 1.0 g or more of aspirated secretions between and within groups at p < 0.05 and power of 80%. It was determined that a minimum of 29 patients per group was required, totaling 87 patients (VB, HMV and VB + HMV), and the sample size was increased by 10% to allow for potential losses during allocation.

### Statistical analysis

The data were analyzed on an intention-to-treat basis using the Statistical Package for the Social Sciences, version 20.0 (SPSS, Inc., Chicago, IL, USA). Quantitative variables with normal distribution were described as means and standard deviations, whereas quantitative variables with nonparametric distribution were described as medians and interquartile ranges, and categorical variables were described as absolute frequencies and percentages. Normal distribution was confirmed using the Shapiro-Wilk test. One-way analysis of variance (ANOVA) was used to compare the distribution of the means of the normal quantitative variables among the groups; for nonparametric medians, the Kruskal-Wallis test was used, and for categorical variables, the chi-square test was used. The Wilcoxon test was used for intragroup comparisons of the medians of the nonparametric variables. For the correction of multiple comparisons, the Tukey test was used, and the level of significance considered was p < 0.05.

## RESULTS

A total of 93 patients (considering losses in allocation) - 29 in the VB group, 32 in the HMV and 32 in the VB + HMV group - were included in the study from February 2012 to November 2014. The characterization of the sample is shown in table 1. There was no significant difference among the groups in terms of the clinical and sociodemographic characteristics studied.

**Table 1:** Clinical and demographic characteristics of the 93 patients studied

	VB	HMV	HMV + VB	p value
Age (years)	63.45 ± 13.69	62.50 ± 13.05	66.20 ± 11.08	0.503
Female sex	48.3	62.5	56.3	
APACHE II	24.08 ± 7.04	24.93 ± 8.59	23.90 ± 6.63	0.861
Pathologies				
COPD	10.3	9.4	10.8	
Bronchopneumonia	37.9	34.4	37.5	
Sepsis	41.4	34.4	40.6	
CHF	10.3	15.6	9.4	
Stroke	0.0	3.1	0.0	
AIDS	6.7	5.3	0.0	
Cancer	6.7	5.3	0.0	
Immunosuppressed	0.0	3.1	0.0	

VB - vibrocompression; HMV - hyperinflation with mechanical ventilator; APACHE II - Acute Physiology and Chronic Health Evaluation II; COPD - chronic obstructive pulmonary disease; CHF - congestive heart failure; Stroke - stroke; AIDS - acquired immunodeficiency syndrome. The results are expressed as the mean and standard deviation, one-way analysis of variance, or %.

**Figure 3:**
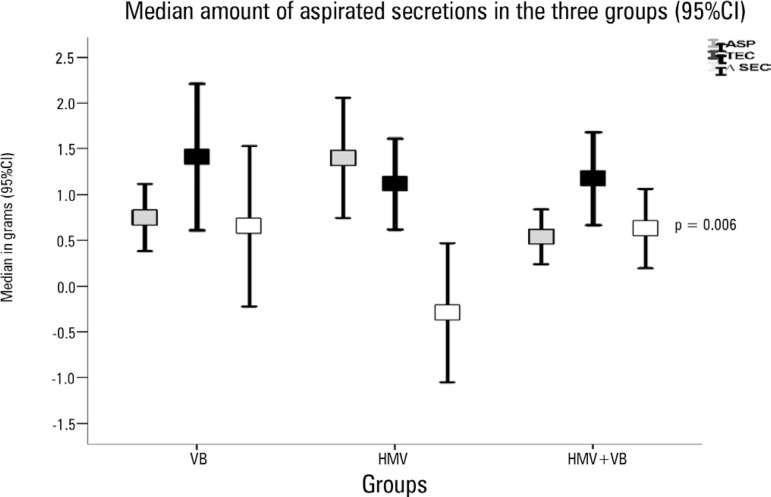
Amount of aspirated secretions, in grams, for pulmonary aspiration alone and each of the studied techniques (vibrocompression, HMV, HMV combined with vibrocompression) and the differences in the amount of aspirated pulmonary secretions (technique minus pulmonary aspiration alone) for the three groups studied. P value obtained by the chi-square test. 95%CI - 95% confidence interval; ASP - pulmonary aspiration alone; TEC - technique; ∆SEC - difference in the amount of aspirated pulmonary secretions (technique minus pulmonary aspiration alone); VB - vibrocompression; HMV - hyperinflation with mechanical ventilator.

Intragroup comparisons (ASP *versus* technique) of the studied sample showed a significant increase in the following parameters after the application of the techniques: HR in all three groups; RR and MAP in the VB group; MAP in the HMV group and TV in the VB + HMV group; however, the differences were not clinically relevant. The other results of the intra- and intergroup comparisons are shown in table 2. The amount of aspirated secretions in grams was higher in the VB + HMV group when compared to ASP, 0.7 g (0.1 - 2.5g) *versus* 0.2g (0 - 0.6g), p = 0.006. However, when comparing the differences in pulmonary secretions (ASP technique) between the groups (VB *versus* HMV *versus* VB + HMV), this result was not statistically significant, according to the graph in figure 3.

**Table 2:** Intragroup comparison of the variation in hemodynamic and pulmonary parameters of the 93 patients studied

Parameters	VB			HMV			HMV + VB		
	ASP	VB	p value	ASP	HMV	p value	ASP	HMV + VB	p value
Heart frequency (bpm)	93 (70 - 110)	101 (75 - 108)	0.006	92 (88 - 99)	94 (88 - 99)	0.022	85 (73 - 98)	83 (70 - 102)	0.553
Respiratory frequency (mrm)	18 (15 - 22)	19 (16 - 22)	0.032	17 (15 - 22)	18 (15 - 22)	0.845	17 (15 - 19)	16 (15 - 18)	0.970
Mean arterial pressure (mmHg)	93 (75 - 101)	82 (72 - 99)	0.001	82 (76 - 99)	84 (73 - 98)	0.045	77 (68 - 93)	78 (66 - 95)	0.164
Peak expiratory pressure (cmH_2_O)	22 (17 - 24)	20 (17 - 24)	0.593	22 (18 - 24)	22 (16 - 24)	0.606	19 (15 - 27)	21 (17 - 25)	0.077
Dynamic compliance (cmH_2_O)	34 (29 - 44)	35 (32 - 51)	0.289	36 (31 - 54)	37 (32 - 47)	0.666	36 (27 - 46)	37 (31 - 47)	0.417
SpO_2_ (%)	99 (97 - 100)	100 (95 - 100)	0.146	98 (94 - 100)	97 (93 - 100)	0.178	99 (97 - 100)	100 (97 - 100)	0.190
Tidal volume (mL)	512 (467 - 613)	522 (462 - 597)	0.214	555 (483 - 664	573 (472 - 638	0.746	490 (450 - 580)	533 (498 - 774)	0.002

VB - vibrocompression; ASP - tracheal aspiration; HMV - hyperinflation with mechanical ventilator; SpO_2_ - peripheral oxygen saturation. The results are expressed as the median and interquartile range (25 - 75%); p < 0.05 Wilcoxon test.

Regarding time and mortality on MV, the three groups showed no significant difference. Regarding the frequency of tracheal reintubation, the HMV group presented an increase when compared to the VB and VB + HMV groups (22.1% *versus* 4.2% *versus* 7.3%), but the difference was not statistically significant (p = 0.083), as shown in table 3.

**Table 3:** Comparison of the time on mechanical ventilation, incidence of death and reintubation within 48 hours in the 93 patients studied

	VB	HMV	HMV+VB	p value
Time on MV (days)	6 (2 - 38)	4.5 (2 - 30)	5 (1 - 16)	0.151
Death	20.7	15.6	18.8	0.507
Reintubation within 48 hours	4.2	22.1	7.3	0.830

VB - vibrocompression; HMV - hyperinflation with mechanical ventilator; MV - mechanical ventilation. The results are expressed as the median (minimum and maximum), Kruskal-Wallis test/Tukey test, or %.

## DISCUSSION

In this study, the use of HMV + VB increased the amount of aspirated pulmonary secretions, a finding that was not observed for the other techniques. In addition, no relevant clinical changes in the hemodynamic and pulmonary parameters were observed for any of the three groups studied. A possible protective effect of HMV + VB and VB on the need for tracheal reintubation within 48 hours was also found.

Previous studies have shown that lung hyperinflation techniques can improve pulmonary oxygenation and compliance and reverse lung collapse and atelectasis.^(^^2,7-10,15^^)^ This is due to the ability of hyperinflation to increase TV, leading to the expansion of both ventilated and collapsed alveoli; this phenomenon is explained by the pulmonary interdependence mechanism, which facilitates the transport of secretions from the peripheral airways to the central airways.^(^^15,16^^)^

The findings of our study regarding the increased amount of aspirated pulmonary secretions after the application of the combined HMV + VB techniques combined were similar to those of Lemes et al., who, in a randomized crossover trial with patients on MV, demonstrated an increase in the amount of pulmonary secretions aspirated after HMV.^(^^17^^)^ Corroborating these findings, our group, in a previous randomized crossover study of patients on MV, also found an increase in the amount of pulmonary secretions aspirated after the combined application of HMV and VB.^(^^5^^)^ However, in these two previous studies, the HMV technique required the application of the patients' ventilatory drive, which made it unfeasible for sedated patients. This disadvantage has been minimized with the current technique since it was performed in assisted-controlled ventilation modes and did not require the patient's ventilatory drive.

The VB technique alone was evaluated by Unoki et al. in a randomized crossover study with patients on MV, and no increase in the amount of aspirated pulmonary secretions was observed.^(^^11^^)^ In addition, in a systematic review, Borges et al. did not find a positive effect on the amount of aspirated secretions in studies that compared VB alone with a control group.^(^^18^^)^ Both studies reinforce our findings. Thus, HMV appears to be a crucial factor in facilitating the aspiration of pulmonary secretions.

Another important factor is that the increased amount of aspirated pulmonary secretion seems to be associated with a decrease in the need for tracheal reintubation. As demonstrated by Gonçalves et al. in a randomized study performed with patients on MV, a tracheal reintubation incidence of 17% was found in the intervention group (pulmonary insufflation-exsufflation bronchial hygiene technique plus usual care) and in 48% in the control group (usual care), p < 0.05. In addition, 5.7% of the patients in the intervention group had secretion retention, *versus* 22.5% in the control group.^(^^19^^)^ Corroborating these findings, Miu et al., in a cohort study with 2,007 patients on MV, reported an increase in the frequency of aspirations in 24 hours (8.4 ± 4.0 *versus* 6.6 ± 4.1) and in the secretion score of reintubated patients compared to those who were not reintubated (12.2 ± 8.0 *versus* 9.0 ± 7.0), with p < 0.01.^(^^20^^)^ In this study, the secretion score was calculated based on the following values over 24 hours: 3 points for a large amount of aspirated pulmonary secretions, two points for intermediate secretions and one point for minimal secretions.

The results of these studies are similar to those found by our group, suggesting that the VB technique, and especially its combination with HMV, can reduce the need for tracheal reintubation, possibly reducing the accumulation of pulmonary secretions by increasing the peak expiratory flow.^(^^21^^)^

This study has some limitations, such as the heterogeneity in the amount of baseline secretions found in the studied sample; additional studies with larger samples are required to demonstrate significant differences between groups. Another limitation was the lack of control of variables that might have influenced the tracheal reintubation outcomes, such as the need for and use of antibiotic therapy, the number of surgical interventions and stratification of the sample by underlying disease.

## CONCLUSION

The hyperinflation with mechanical ventilation technique combined with vibrocompression, when compared to aspiration alone, is more effective for the removal of secretions as evidenced by the increased amount of aspirated pulmonary secretions. Further studies are needed to better detail the physiological mechanism underlying the outcomes of these bronchial hygiene techniques to further clarify their effect on patients on mechanical ventilation.
